# *In vitro* and *in vivo* antibacterial efficacy of bacteriophage combined with tigecycline against carbapenem-resistant *Klebsiella pneumoniae* and characterization of phage resistant mutants

**DOI:** 10.3389/fcimb.2025.1610625

**Published:** 2025-09-04

**Authors:** Rui Zhu, Ruilin Wang, Bing Fei, Ruici Lu, Xiaojuan You, Xinwei Liu, Chunxia Wang, Yongwei Li

**Affiliations:** ^1^ Henan Province Hospital of Traditional Chinese Medicine (The Second Affiliated Hospital of Henan University of Chinese Medicine), Zhengzhou, China; ^2^ The Second Clinical Medical College, Henan University of Chinese Medicine, Zhengzhou, China; ^3^ The Key Laboratory of Pathogenic Microbes and Antimicrobial Resistance Surveillance of Zhengzhou, Henan Province Hospital of Traditional Chinese Medicine, Zhengzhou, China; ^4^ Henan Engineering Research Center for Identification of Pathogenic Microbes, Henan Province Hospital of Traditional Chinese Medicine, Zhengzhou, China; ^5^ Henan Provincial Key Laboratory of Antibiotics-Resistant Bacterial Infection Prevention and Therapy with Traditional Chinese Medicine, Henan Province Hospital of Traditional Chinese Medicine, Zhengzhou, China

**Keywords:** carbapenem-resistant Klebsiella pneumoniae, phage therapy, biofilm, phage resistance, Galleria mellonella infection model

## Abstract

Carbapenem-resistant *Klebsiella pneumoniae* (CRKP) has emerged as a critical global public health threat, characterized by high infection rates, elevated mortality, and limited therapeutic options. In this study, we isolated and characterized a novel bacteriophage (phage), designated as HZJ31, which exhibited potent lytic activity against CRKP strains. Phylogenetic and genomic analyses revealed that phage HZJ31 belongs to the order *Caudovirales* and lacks virulence factors, antibiotic resistance genes, and lysogeny-related elements, supporting its suitability for therapeutic applications. Phage HZJ31 exhibits remarkable anti-biofilm activity by preventing biofilm formation and disrupting established biofilms, with bacterial reduction rates exceeding 70% (*P*<0.05). In combination with Tigecycline, it significantly enhanced bactericidal efficacy, delayed the emergence of phage resistant mutants, and improved survival rates in *Galleria mellonella* larvae infection models. Compared to the bacterial-infected group, which had 80% larval mortality at 96 h, treatment with HZJ31 or TGC alone led to 50% and 60% survival, while their combination improved survival to 70% (*P* < 0.05). Notably, the phage-resistant mutant, which emerged due to capsule loss, resulted in reduced growth and virulence, while regaining sensitivity to certain antibiotics (such as gentamicin), indicating a fitness cost associated with phage resistance. Collectively, these findings provide valuable insights into phage-antibiotic synergy and underscore the promising clinical potential of phage HZJ31 as a therapeutic agent against CRKP infections.

## 1 Introduction

Antibiotic resistance is acknowledged as a critical threat to public health. Carbapenems, including imipenem and meropenem, are highly effective broad-spectrum β-lactam antibiotics used as a last resort against multidrug-resistant Gram-negative infections, particularly those caused by ESBL- and AmpC-producing *Klebsiella pneumoniae* (KP) strains (Bologna et al., 2024; Naghavi et al., 2024). The extensive clinical utilization of carbapenems has led to the global rise of carbapenem-resistant *Klebsiella pneumoniae* (CRKP) (Ding et al., 2023; Hu et al., 2024a). In 2024, according to the China Antimicrobial Surveillance Network (CHINET), KP strains ranked second among all clinical isolates, accounting for 13.9% (n=458271), second only to *Escherichia coli* at 18.2%. From 2014 to 2024, clinical KP isolates showed increased resistance to imipenem and meropenem, rising from 11.0% and 14.1% to 22.6% and 23.4%, respectively, indicating a concerning trend (Karampatakis et al., 2023). Infections with CRKP endure a significant fatality rate and are challenging to treat (Lou et al., 2022; Hu et al., 2024a). In 2017, WHO published the WHO Priority Pathogens List for R&D of New Antibiotics, where carbapenem-resistant Enterobacteriaceae (including CRKP) was categorized as a “Critical” priority level (Miller and Arias, 2024). Treatment options for CRKP infections are extremely restricted, emphasizing the urgent need for novel antibacterial strategies (Hu et al., 2024a; Xing et al., 2025).

Bacteriophages (phages) are viruses that specifically infect bacteria and cause lysis, characterized by their ubiquity, abundance, diversity, structural simplicity, and amenability to genetic manipulation. In light of the escalating global antimicrobial resistance crisis, phage therapy has garnered renewed interest from the scientific community (Goodridge, 2010; Jault et al., 2019; Patpatia et al., 2021; Uyttebroek et al., 2022). Phages are classified into lytic and lysogenic phages based on their life cycle and replication characteristics. Lytic phages exhibit specific adsorption to bacterial cells and release progeny phages through a five-stage process: adsorption, penetration, biosynthesis, maturation, and release (Suttle, 2007). Bacteriophages, whether administered as monotherapy or in combination regimens, have demonstrated potent bactericidal activity against KP strains (Martins et al., 2022; Mulani et al., 2022), emerging as a promising therapeutic modality for CRKP infections (Baqer et al., 2022; Gorodnichev et al., 2023; Kou et al., 2024). Phage therapy offers distinct advantages over antibiotics, such as effectiveness against multidrug-resistant bacteria, anti-biofilm capabilities, high specificity, potent bactericidal activity, and the potential for genetic engineering (Kortright et al., 2019; Xing et al., 2025). Phage therapy faces multiple challenges, including a restricted host range, the rapid emergence of phage-resistant mutants, possible clearance by the host immune system, the absence of standardized manufacturing protocols and robust clinical trial data (Strathdee et al., 2023) (Uyttebroek et al., 2022). Moreover, recent studies have highlighted the potential risk of horizontal gene transfer (HGT) mediated by phages, which may unintentionally contribute to the dissemination of virulence factors and antibiotic resistance genes among pathogenic bacteria (Colavecchio et al., 2017; Cui et al., 2024; Cook and Hynes, 2025; She et al., 2025). These limitations significantly impede its broader clinical application.

This study identified a newly isolated lytic phage, HZJ31, targeting CRKP, and evaluated its biology, genome, anti-biofilm activity, and antibacterial efficacy *in vitro* and *in vivo*, highlighting its potential as a novel antibacterial strategy for CRKP infections. Notably, the analysis of the phage-resistant strain sheds light on the dynamic interactions between phages and bacteria, while also offering valuable insights into the fitness costs associated with phage resistance.

## 2 Materials and methods

### 2.1 Bacterial strains and culture conditions

Clinical KP strains, including KPZ2 and its phage-resistant mutant KPZ2-R, were isolated from the clinical laboratory at Henan Provincial Hospital of Traditional Chinese Medicine and preserved in 25% glycerol at -80°C. The strains were identified using the IVD MALDI Biotyper mass spectrometer (Bruker Daltonik, Germany). Antimicrobial susceptibility testing was performed using the VITEK 2 Compact system (bioMérieux, France), and results were interpreted according to the Clinical and Laboratory Standards Institute (CLSI) guidelines. The strains were revived on blood agar plates and incubated overnight at 37°C with 5% CO_2_. After that, one colony was added to Luria-Bertani broth and cultured at 37°C with shaking at 180 rpm until the OD600 reached 0.6–1.0, indicating the logarithmic growth phase.

### 2.2 Phage isolation and purification

Phages were isolated from untreated hospital wastewater using a modified enrichment protocol (Wang et al., 2024). To screen for lytic phages, several clinical KP isolates, including KPZ2, were randomly selected as candidate host strains. After being stored at 4°C for 2 h, untreated sewage samples were concentrated at 8000 rpm for 10 min and filtered through a 0.22 μm filter. The filtered sewage was then mixed with an equal volume of 2× LB broth and inoculated with a clinical isolate (OD600 = 0.5). The mixture was incubated overnight at 37°C with shaking at 120 rpm. The phage stock was obtained by collecting the supernatant after centrifuging and filtering the supernatant through a 0.22 μm filter. The double-layer agar plate approach was used to purify phages until a single type of phage morphology was observed.

### 2.3 Transmission electron microscopy

The agarose gel blocks containing phages were incubated in PBS at 4°C for 6 h, then centrifuged and filtered through a 0.22 μm filter to obtain phage suspension. The phage suspension was concentrated with a 30 kDa ultrafiltration tube at 3000 × g and 4°C for 15 min. Phages were adsorbed onto the 200-mesh carbon-coated copper grid and stained with 2% phosphotungstic acid. Phage morphology was observed using a Hitachi HT7800.

### 2.4 Optimal multiplicity of infection

The optimal MOI for phage HZJ31 was determined with a modified protocol (Wang et al., 2024) by incubating it with KPZ2 at MOI values of 0.001, 0.01, 0.1, and 1 at 37°C with shaking at 200 rpm for 4 h. The mixture of cultures was centrifuged at 6000 rpm for 15 min and the supernatant passed through a 0.22 μm filter to remove bacterial cells. Phage titers were determined using the double-layer agar method.

### 2.5 One-step growth curve

The one-step growth curve of phage HZJ31 was evaluated using the method (Fei et al., 2023). Briefly, phages HZJ31(MOI=0.001) and KPZ2 were incubated at 37°C for 15 min to allow adsorption. The mixture was then centrifuged at 12,000 rpm for 5 min at 4°C, and the supernatant was removed. The pellet was resuspended in LB broth and incubated at 37°C with shaking at 200 rpm for 100 min. The samples were collected at 10-minute intervals, and phage titers were quantified to generate the growth curve. The burst size = (Final phage titer - Initial free phage titer​)/Number of infected bacterial cells.

### 2.6 Host range of phage

The host range of phage HZJ31 was determined by the spot assay according to the protocol described previously (Wang et al., 2024). Clinical CRKP isolates were adjusted to an OD600 of 0.5 using PBS, and the bacterial suspensions were evenly spread on LB agar plates. Then, 10 μL of phage HZJ31 was immediately spotted onto the surface, followed by overnight incubation at 37°C. The host range was evaluated through plaque formation: ++ denotes clear plaques, + signifies faint plaques, and - indicates the absence of plaque. All experiments were conducted in triplicate for consistency.

### 2.7 The stability for temperature and pH

The stability of phage was carried out according to the published paper (Fei et al., 2023). The thermal stability of phage HZJ31 was tested by incubating the phage (1×10^10^ PFU/mL) at temperatures of 4, 25, 37, 50, 60, 70, and 80°C for 1 h. For pH stability, phage HZJ31 was mixed with SM buffer at pH values of 3, 5, 7, 9, 10, 11, and 13 in a 1:10 (v/v) ratio and incubated at 37°C for 1 h. Then, phage titers were determined.

### 2.8 Cytotoxicity of phage by CCK-8 assay

The procedures were performed according to the previous study with minor modifications (Wang et al., 2024). The cells were plated at a density of 1×10^4^ cells per well in a 96-well plate and differentiated with 200 ng/mL of phorbol 12-myristate 13-acetate (PMA) overnight at 37°C with 5% CO_2_. Phage HZJ31 was diluted from 10^8^ to 10^14^ PFU/mL in RPMI-1640 with 10% FBS and incubated with cells at 37°C, 5% CO_2_ for 24, 48, and 72 h. Following incubation, 100 μL of a CCK-8 premix (Beijing Solarbio, China) with 90% PBS was added to each well after the cells were washed twice with PBS. After an additional incubation for 3 h, absorbance at OD450 nm was detected.

### 2.9 Genomic extraction of phages

The Phage DNA was extracted utilizing the Viral Gene Extraction Kit (Takara Bio, China). A solution containing 200 μL Buffer VGB, 20 μL Proteinase K, one μL Carrier RNA, and 200 μL phage suspension (10^8^ PFU/mL) was incubated at 56°C for 10 min.200 μL of anhydrous ethanol was added and mixed before transferring to a spin column. The mixture was centrifuged at 12,000 rpm for 2 min, and the filtrate was discarded. Subsequently, 500 μL of Buffer RWA and 700 μL of Buffer RWB were added in sequence, centrifuged, and discarded, with Buffer RWB applied twice, followed by centrifugation at 12,000 rpm for 3 min. The spin column was then transferred to a new RNase-free tube, and 60 μL of RNase-free ddH2O was added to elute the DNA. This mixture was incubated for 5 min and centrifuged at 12,000 rpm for 5 min to collect the phage DNA.

### 2.10 Phage genome sequencing and annotation

The phage genome was sequenced using the Illumina HiSeq 2500 platform, yielding a high average sequencing depth of 24,068×. Raw reads were filtered and quality-checked using *fastp*, and *de novo* assembly was performed with *SPAdes*. The quality of the assembly was evaluated using *QUAST*, resulting in an N50 value of 40,624 bp, indicating good assembly continuity. The genome was uploaded to the National Center for Biotechnology Information (NCBI). It was then analyzed with BLASTn to identify similar phages in the GenBank database. The tRNA genes encoded by the phage were identified using tRNAscan-SE. Phage-encoded genes were annotated using the Prokaryotic Genome Annotation System (Prokka). Functional characterization of the predicted amino acid sequences of these genes was conducted using NCBI’s BLASTp. Virulence factors and antibiotic resistance genes were identified using the VFDB and ARDB databases (thresholds of ≥70% identity and ≥70% coverage). The genomic map was visualized with CGview.

### 2.11 Phylogenetic analysis of phage HZJ31

The terminase large subunit sequences were used for phylogenetic analysis. The amino acid sequence of terminase large subunit of phage HZJ31 was entered into NCBI’s BLASTp, resulting in the identification of ten homologous sequences with notable similarity. The basis for generating a phylogenetic tree using the ClustalW alignment tool within the MEGA 11.0 platform. Furthermore, the genome sequences of phage HZJ31 and the selected phage were analyzed by Easyfig.

### 2.12 Determination of minimum inhibitory concentration

The MICs of tigecycline (TGC) against KPZ2 were conducted by the broth dilution method. TGC was serially diluted in LB broth in twofold dilutions.100 μL of each antibiotic dilution was added to the corresponding wells of a 96-well plate. Negative control wells contained only medium without bacteria, while positive control wells contained bacteria without antibiotics. The KPZ2 bacterial suspension was diluted in LB broth to 5 × 10^5^ CFU/mL from an initial McFarland 0.5 suspension. Then, 100 μL of the bacteria was added to each well. The plate was incubated for 18 h at 37°C. The MIC was determined as the lowest concentration of TGC that inhibited visible bacterial growth.

### 2.13 Bactericidal kinetics of the combined phage HZJ31 and tigecycline

The assay was performed according to a previously reported method (Wang et al., 2024), with minor modifications. The experimental design included the following groups: The KPZ2 group, The phage HZJ31 group (MOI = 0.001), The 1/2 MIC TGC group, and the phage HZJ31 with 1/2 MIC TGC group. The KPZ2 was standardized to 1×10^6^ CFU/mL. 100 μL of the bacterial culture was added to the 96-well plate, followed by the addition of phage HZJ31 and/or TGC, according to group assignment. The plate was incubated at 37°C for 36 h, with OD600 measurements taken hourly to construct bactericidal kinetics curves to evaluate the inhibitory effects of phage and/or TGC on bacterial growth. Synergy was defined as a statistically significant enhancement of antibacterial activity in the combination group (phage + TGC) compared to each monotherapy group, based on OD600 values measured over time. Statistical analysis was performed using two-way ANOVA followed by Tukey’s multiple comparison test (*P* < 0.05).

### 2.14 Antibacterial efficacy of phage HZJ31 and TGC in the *Galleria mellonella* infection model

The experiments were evaluated using a *Galleria mellonella*(*G.mellonella*)larvae infection model using a protocol modified from a previous study (Peng et al., 2025). The larvae, approximately 2–3 cm in length and weighing 250–300 mg, with a uniform milky white color and no gray spots, exhibiting high vitality, were selected for the experiments (purchased from Tianjin Huiyude Biotechnology Co., Ltd., China). To determine the optimal bacterial infection dose, we selected the concentration that resulted in approximately 80% larval mortality at 24 h post-infection as the target inoculum. Before experimentation, larvae were acclimated at 37°C for 1–2 h, and KPZ2 was cultured in LB broth to reach an OD600 of 0.8-1.0. The culture underwent centrifugation at 7,000 rpm for 10 min, followed by PBS washing and adjustment to concentrations of 10^5^ to 10^9^ CFU/mL. 10 µL bacterial suspension or PBS (as control) was administered into the last left proleg of the larvae. Larvae were incubated at 37°C with survival rates monitored every 12 h.

To evaluate the combined antibacterial efficacy of phage HZJ31 and TGC in the *G.mellonella* infection model, larvae were randomly assigned to five groups: the PBS control group, the KPZ2 group, the phage HZJ31 group, the TGC group, and the phage HZJ31 + TGC group. Except for the PBS control group (injected with an equal volume of PBS), each larva received 10 μL of KPZ2 suspension (at a concentration determined by preliminary experiments) into the last left proleg. After 1 h, 10 μL of the respective treatment was injected into the last right proleg of each larva: PBS (for the PBS control and KPZ2 group), phage HZJ31 (MOI = 0.001, HZJ31 group), 1/2 MIC TGC (TGC group), and a mixture of phage HZJ31 and 1/2 MIC TGC (combined treatment group). All larvae were incubated at 37°C and monitored every 12 h for 4 consecutive days. Survival status was recorded, and survival rates were calculated for each group to evaluate therapeutic efficacy.

### 2.15 Biofilm quantification by crystal violet assay

The biofilm quantification was performed using a modified protocol

(Wang et al., 2024). A 96-well plate was incubated at 37°C for 24 h, 48 h, and 72 h with KPZ2 suspension (10^6^ CFU/mL) and phage HZJ31 (MOI =0.001). For biofilm disruption, another set of KPZ2 cultures was incubated for 48 h to form biofilms, which were then treated with phage HZJ31 (MOI = 0.01) for 6 h. After washing with PBS to remove planktonic cells, biofilms were fixed with methanol for 30 min and stained with 0.1% crystal violet solution (Shanghai Baisai, China) for 15 min. The residual stain was removed by PBS washing, and biofilm biomass was quantified by dissolving crystal violet in 200 μL of 30% glacial acetic acid. Absorbance at 595 nm was measured to assess biofilm biomass. All experiments were performed in triplicate.

### 2.16 Bactericidal activity in biofilms by XTT assay

The experiment was conducted with suitable modifications (Fei et al., 2023). KPZ2 suspension (10^6^ CFU/mL) and phage HZJ31 (MOI = 0.001) were co-incubated in a 96-well plate at 37°C for 24 h, 48 h, and 72 h. For biofilm disruption, preformed 48 h biofilms were treated with HZJ31 (MOI = 0.01) for 6 h. After incubation, PBS was used to wash the plate to remove planktonic bacteria. Subsequently, 200 μL of LB broth and 20 μL of XTT solution (Shanghai Yuanye, China) were introduced into each well. The plate was then incubated at 37°C in the dark for 3 h, followed by absorbance measurement at OD490 nm. The inhibitory rate was calculated using the following formula:

$[1-\frac{A\left(\text{Experimental group}\right)-A\left(\text{Blank control group}\right)}{A\left(\text{Control group}\right)-A\left(\text{Blank control group}\right)}]\times 100\%.$

The experiment was repeated three times.

### 2.17 Screening of phage-resistant bacteria

The experiment was performed with some modifications based on a previous study (Wang et al., 2024). KPZ2 and phage HZJ31 (MOI =0.001) were incubated at 37°C with shaking at 200 rpm for 24 h. Cultures were streaked onto blood agar plates to isolate single colonies. The isolates were identified by the mass spectrometer. Phage resistance was confirmed by spot testing and double-layer agar assays. Resistant strains were designated KPZ2-R.

### 2.18 Scanning electron microscopy

KPZ2 and KPZ2-R were incubated at 37°C with shaking at 220 rpm for 6 h. The cultures were then centrifuged at 8000 rpm for 10 min to collect the pellets, which were subsequently washed with PBS. Pellets were fixed in 2.5% glutaraldehyde at room temperature for 5 h, followed by PBS washing and 15-minute dehydration using graded ethanol concentrations (30%, 50%, 70%, 90%, 100%). After dehydration, the samples were dried, gold-coated, and observed under the SEM Hitachi SU8100 to capture morphology.

### 2.19 Antibiotic susceptibility testing

Antibiotic susceptibility of KPZ2 and KPZ2-R was determined using the VITEK-2 Compact system (BioMérieux, France). The KPZ2 and KPZ2-R were standardized to a 0.5 McFarland scale. The prepared bacterial suspension was loaded along with the AST-GN card into the VITEK-2 Compact system for automatic inoculation and determination. The results were analyzed following the guidelines of the Clinical and Laboratory Standards Institute (CLSI).

### 2.20 Phage adsorption efficiency

The assay was performed using a modified protocol (Wang et al., 2024). KPZ2 and KPZ2-R were incubated with phage HZJ31 (MOI =0.001) at 37°C, with samples collected at 0, 5, 10, 15, and 20 min. The samples were centrifuged at 11000 rpm for 8 min at 4°C. The supernatants were filtered (0.22 μm), and then the phage titer was determined. The Adsorption Efficiency = [(Initial Phage Titer - Phage Titer in Supernatant)/Initial Phage Titer] × 100%.

### 2.21 Bacterial growth rates

KPZ2 and KPZ2-R were adjusted to 1.0 × 10^6^ CFU/mL. The 96-well plate was prepared with 200 μL of bacterial suspension in each test well and 200 μL of LB broth in the control wells. The plate was incubated at 37°C for 24 h with hourly OD600 measurements.

### 2.22 Comparison of virulence using the *G.mellonella* infection model

The concentrations of KPZ2 and KPZ2-R were adjusted to 10^6^ and 10^7^ CFU/mL, respectively. Each *G.mellonella* larvae received an injection of 10 µL bacterial suspension (PBS as a control) into the last left proleg. Larvae were incubated at 37°C with survival monitored every 12 h over 4 days.

### 2.23 Bacterial whole-genome sequencing

Genomic DNA of strains KPZ2 and KPZ2-R was extracted using a whole-genome extraction kit (Sangon Biotech, China) according to the manufacturer’s instructions. Briefly, 5 mL of overnight bacterial culture was centrifuged to collect the cells, followed by the addition of lysis buffer and incubation at 65 °C for 1 h to lyse the cells. Subsequently, Buffer PB was added, mixed thoroughly, and briefly incubated at −20 °C. After centrifugation, the supernatant was transferred, isopropanol was added, mixed well, and centrifuged again to pellet the DNA. The DNA pellet was washed with 75% ethanol, dried after removing the ethanol, and then dissolved in TE buffer. DNA concentration and integrity were assessed using a Qubit 4.0 fluorometer (Thermo Fisher Scientific, China). The extracted DNA was either used for subsequent experiments or stored at −20°C.

The genomes of strains KPZ2 and KPZ2-R were sequenced using the BGI MGISEQ 2000 platform. The extracted DNA was enzymatically fragmented, end-repaired, ligated with adaptors, and amplified by PCR to construct sequencing libraries. Library concentration was quantified using a Qubit fluorometer, and fragment size distribution was assessed using the Agilent 2100 Bioanalyzer; libraries with a main peak around 500 bp were considered qualified. The libraries were then denatured, circularized, and purified before sequencing. Raw sequencing data were subjected to quality control to remove adaptors and low-quality reads. The filtered reads were assembled using SPAdes (v3.13.0). Based on alignment results, ANGSD (v0.940) was used to reconstruct the genome sequences, and genome completeness and contamination were evaluated using CheckM (v1.2.2).

The K-locus types of KPZ2 and KPZ2-R were identified using the Kaptive Web tool (https://kaptive-web.erc.monash.edu/). Genome assemblies in FASTA format were uploaded, and typing was based on sequence coverage, identity, and completeness. Confidence levels were recorded for each result.

### 2.24 Statistical analysis

All statistical analyses were conducted using GraphPad Prism 9.2. One-way or two-way analysis of variance (ANOVA) followed by Tukey’s multiple comparisons test was employed to undertake multiple group comparisons. Survival curves for *G.mellonella* were analyzed using the log-rank (Mantel-Cox) test. Statistical significance was indicated by *P <0.05, **P <0.01, ***P < 0.001, and ****P <0.0001.

## 3 Results

### 3.1 Isolation and characterization of phage HZJ31

**Figure 1:**
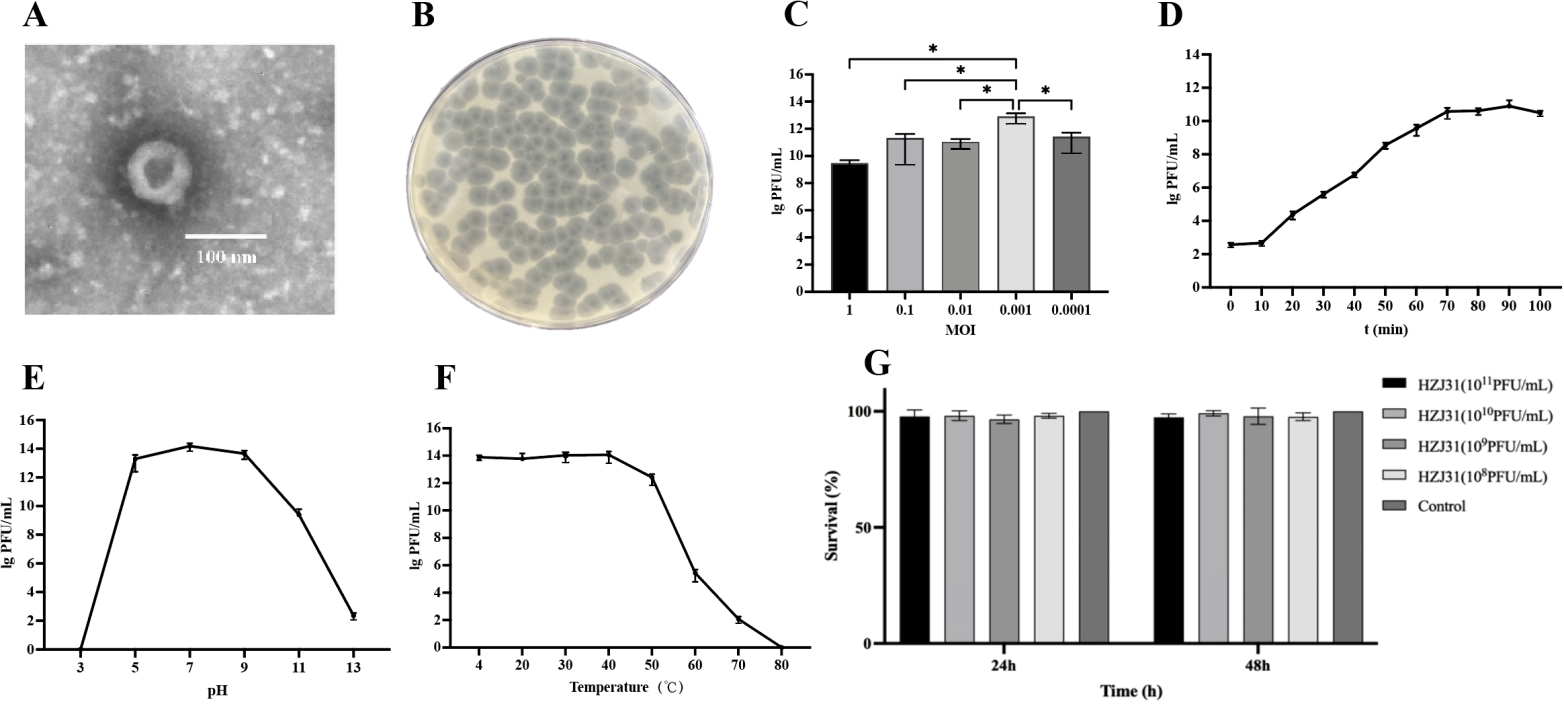
Biological characteristics, stability and cytotoxicity of phage HZJ31. **(A)** TEM morphology of phage HZJ31, revealing an icosahedral head (~80 nm) and short tail (~10 nm), consistent with *Caudovirales*. **(B)** Plaque morphology of phage HZJ31 on a double-layer agar plate. **(C)** The optimal MOI of phage HZJ31, the highest titer was observed at MOI = 0.001, mean ± SD. **(D)**The one-step growth curve of phage HZJ31, mean ± SD. **(E)** The pH stability of phage HZJ31, the phage remained stable between pH 5–9 but was inactivated under extreme acidic or alkaline conditions. mean ± SD. **(F)** Thermal stability of phage HZJ31. phage activity was maintained up to 50 °C and declined at higher temperatures, with complete inactivation at 80 °C. mean ± SD. **(G)** Cytotoxicity assessment of phage HZJ31 on THP-1 cells by CCK-8 assay. no significant cytotoxicity was observed after 24 h and 48 h exposure to phage HZJ31 at various concentrations (10^8^ to 10^11^ PFU/mL). mean ± SD. *P < 0.05.

Phage HZJ31 was isolated using strain KPZ2 as the host bacterium from wastewater. TEM revealed the phage had an icosahedral head approximately 80 nm in diameter and a short tail measuring 10 nm (**Figure 1A**). The plaques were large, clear, and uniform, reaching up to 4.0 mm in diameter, with a halo of approximately 7.2 mm, which indicates the high efficiency (**Figure 1B**). Phage HZJ31 was classified within the *Caudovirales* based on its morphological characteristics.

The double-layer agar plate method was employed to determine the optimal MOI, one-step growth, and stability of phage HZJ31. Phage titer of HZJ31 remained largely stable across MOIs from 0.1 to 0.0001, peaking at an MOI of 0.001, which was chosen for further experiments (**Figure 1C**). The one-step growth curve showed a 10-minute latent period, followed by a rapid exponential rise in phage population between 10 and 70 min, culminating in a plateau phase. The estimated burst size was 374 PFU/cell, suggesting that HZJ31 possesses a highly efficient host cell lysis capability (**Figure 1D**).

The environmental stability of phage HZJ31 was further assessed under varying pH and temperature conditions. Phage HZJ31 exhibited stable titers between pH 5 and 9, with the highest stability observed around neutral pH. In contrast, the phage titer drastically decreased under highly alkaline (pH > 9) and acidic (pH < 5) conditions, becoming nearly undetectable at pH 3 (**Figure 1E**). The thermal stability of HZJ31 was robust between 4°C and 50°C, but its titer began to decrease beyond 50°C, and complete inactivation occurred at 80°C (**Figure 1F**). The stability of HZJ31 indicates its potential suitability for phage therapy applications. Phage HZJ31 demonstrates strong host specificity, with a lysis rate of 23.33% (7/30) against clinical CRKP isolates and only 13.33% (4/30) achieving complete lysis (**Table 1**). Furthermore, CCK-8 assays showed that co-culturing THP-1 cells with HZJ31 at concentrations ranging from 10^8^ to 10^11^ PFU/mL for 24 h and 48 h did not significantly affect cell viability compared to the untreated control (mean ± SD, n=6, **Figure 1G**), confirming the cellular safety of HZJ31 for therapeutic applications.

**Table 1:** The host range of phage HZJ31.

Strain	Type	Source	Lysis capability
KPZ2	CRKP	Sputum	++
154	CRKP	Sputum	–
156	CRKP	Sputum	–
159	CRKP	Sputum	–
160	CRKP	Ascites	–
168	CRKP	Urine	–
172	CRKP	Sputum	–
173	CRKP	Pus secretion	–
294	CRKP	Sputum	+
302	CRKP	Rectal Swab	–
303	CRKP	Urine	+
309	CRKP	Sputum	+
312	CRKP	Sputum	–
314	CRKP	Sputum	–
316	CRKP	Rectal Swab	–
318	CRKP	Sputum	–
322	CRKP	Sputum	–
323	CRKP	Sputum	–
328	CRKP	Rectal Swab	–
329	CRKP	Sputum	–
341	CRKP	Urine	–
344	CRKP	Sputum	–
488	CRKP	Sputum	++
662	CRKP	Sputum	–
665	CRKP	Urine	–
**682**	CRKP	Sputum	**-**
692	CRKP	Sputum	–
693	CRKP	Blood	–
L8	hv-CRKP	Sputum	++
L9	hv-CRKP	Blood	++

‘++’ denotes clear plaques, ‘+’ signifies faint plaques, and ‘-’ indicates the absence of plaque

### 3.2 Genomic analysis of phage HZJ31

**Figure 2:**
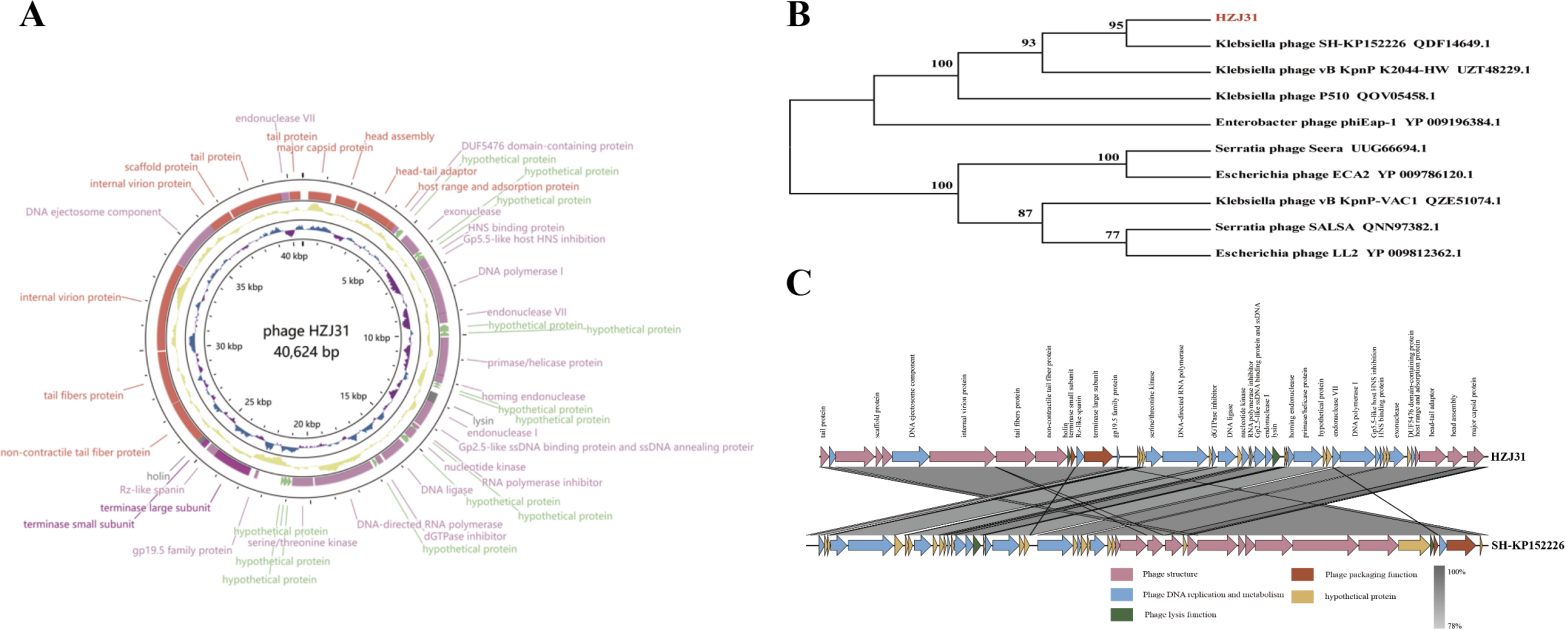
Genomic analysis of phage HZJ31. **(A)** Gene map of Phage HZJ31. **(B)** Phylogenetic tree phage HZJ31 based on terminase large subunit. **(C)** Genomic comparative of phage HZJ31 and *Klebsiella* phage SH-KP152226.

The genome of Phage HZJ31 (GenBank: OR050820.1) was sequenced using Illumina HiSeq 2500 platform, identifying a circular dsDNA molecule of 40,624 bp with a GC content of 53.16%. The genomic map was constructed using GCview (**Figure 2A**). Annotation using Prokka identified 48 ORFs, of which 33 were functionally annotated and classified into four major categories: structural proteins, DNA replication and metabolism, lysis mechanisms, and genome packaging (**Table 2**). In the structural module, ORF1 encodes a major capsid protein (98.84% identity with phage IME264), and ORF4 encodes a host adsorption protein (100% identity with phage K11), both essential for host recognition and infection. Tail-associated proteins such as ORF40 and ORF41 (tail fiber proteins) contribute to phage structural assembly. Replication-related genes include ORF12 (DNA polymerase I) and ORF16 (primase/helicase), while ORF30 encodes a DNA-directed RNA polymerase, suggesting the phage can replicate and transcribe independently of the host. Lysis functions are attributed to ORF20, encoding an amidase/lysin, and ORF39, encoding a holin, which together facilitates host cell wall degradation and phage release. Packaging proteins ORF36 and ORF38 encode the large and small terminase subunits, respectively, responsible for DNA cleavage and encapsidation. Overall, HZJ31 harbors a complete set of functional modules characteristic of a lytic phage, supporting its potential as a therapeutic agent against CRKP infections.

**Table 2:** Functional gene annotation of phage HZJ31 (GenBank:OR050820.1).

Function	No.	Start position	Stop position	Strand	Predicted function	Similar species	Identified
Phage structure	ORF1	1288	254	–	major capsid protein	Klebsiella phage IME264	98.84%
	ORF2	2466	1516	–	head assembly	Klebsiella phage VLCpiA3d	96.84%
	ORF3	4177	2570	–	head-tail adaptor	Klebsiella phage K5	98.50%
	ORF4	4449	4189	–	host range and adsorption protein	Klebsiella phage K11)	100%
	ORF40	27480	25555	–	non-contractile tail fiber protein	Klebsiella phage P560	98.44%
	ORF41	29880	27499	–	tail fibers protein	Klebsiella phage IME304	98.99%
	ORF42	33908	29943	–	internal virion protein	Klebsiella phage vB_KpnP_ZK2	98.86%
	ORF44	36770	36180	–	internal virion protein	Klebsiella phage KN4-1	100%
	ORF45	37183	36773	–	scaffold protein	Klebsiella phage P560	100%
	ORF46	39632	37257	–	tail protein	Klebsiella phage 2044-307w	97.85%
	ORF48	40516	40022	–	tail protein	Klebsiella phage vB_KpnP_IME205	99.39%
Phage DNA replication and metabolism	ORF5	4672	4451	–	DUF5476 domain-containing protein	Klebsiella phage vB_KpnP_IME205	100%
	ORF7	6005	5100	–	exonuclease	Klebsiella phage vB_KpnP_IME205	99.34%
	ORF10	6583	6374	–	HNS binding protein	Klebsiella phage vB_Kp1	100%
	ORF11	6864	6580	–	Gp5.5-like host HNS inhibition	Klebsiella phage SH-Kp 152410	95.74%
	ORF12	9009	6883	–	DNA polymerase I	Klebsiella phage vB_KpnP_BIS33	96.75%
	ORF13	9442	8990	–	endonuclease VII	Klebsiella phage vB_KpnP_BIS33	98.00%
	ORF16	11818	10100		primase/helicase protein	Klebsiella phage NL_ZS_1	99.48%
	ORF17	12158	11793		homing endonuclease	Klebsiella phage IME304	100%
	ORF21	13542	13093	–	endonuclease I	Klebsiella phage KMI1	98.66%
	ORF22	14237	13542	–	Gp2.5-like ssDNA binding protein and ssDNA annealing protein	Klebsiella phage vB_KpnP_IME205	100%
	ORF23	14446	14297	–	RNA polymerase inhibitor	Klebsiella phage K11	91.84%
	ORF25	14955	14527	–	nucleotide kinase	Klebsiella phage vB_KpnP1	97.18%
	ORF27	16410	15328	–	DNA ligase	Klebsiella phage Tokugawa	97.50%
	ORF28	16768	16511	–	dGTPase inhibitor	Klebsiella phage VLCpiA3b	97.65%
	ORF30	19768	17048	–	DNA-directed RNA polymerase	Klebsiella phage vB_KpnP_FZ12	99.01%
	ORF31	20799	19840	–	serine/threonine kinase	Enterobacter phage ENC14	86.35%
	ORF43	36180	33925	–	DNA ejectosome component	Klebsiella phage P560	99.20%
	ORF47	39999	39598	–	endonuclease VII	Klebsiella phage KP32_isolate 195	95.49%
Phage lysis function	ORF20	13090	12635	–	amidase/lysin	Klebsiella phage vB_Kp_IME531	95.36%
	ORF39	25545	25342	–	holin	Klebsiella phage VLCpiA3a	100%
Phage packaging	ORF36	24541	22784	–	terminase large subunit	Klebsiella phage P560	99.83%
	ORF38	25338	25081	–	terminase small subunit	Klebsiella phage vB_KpnP_KpV767	98.82%

The absence of virulence or antibiotic resistance genes in the VFDB and ARDB databases highlights the phage’s biosafety for therapeutic use. Genomic analysis using BLASTn revealed that phage HZJ31 exhibits 94.84% sequence identity with Klebsiella phage 066012 (GenBank: MW042787.1) with 94% coverage. The terminase large subunit gene of HZJ31 was found to be highly conserved, and phylogenetic analysis based on this gene positioned HZJ31 in close relation to Klebsiella phage SH-KP152226 (QDF14649.1) (**Figures 2B, C**). These findings collectively indicate that HZJ31 represents a novel Klebsiella-specific phage, expanding our understanding of phage diversity and potential for therapeutic exploitation.

### 3.3 Antibacterial efficacy of phage HZJ31 and TGC *in vitro* and *in vivo*

**Figure 3:**
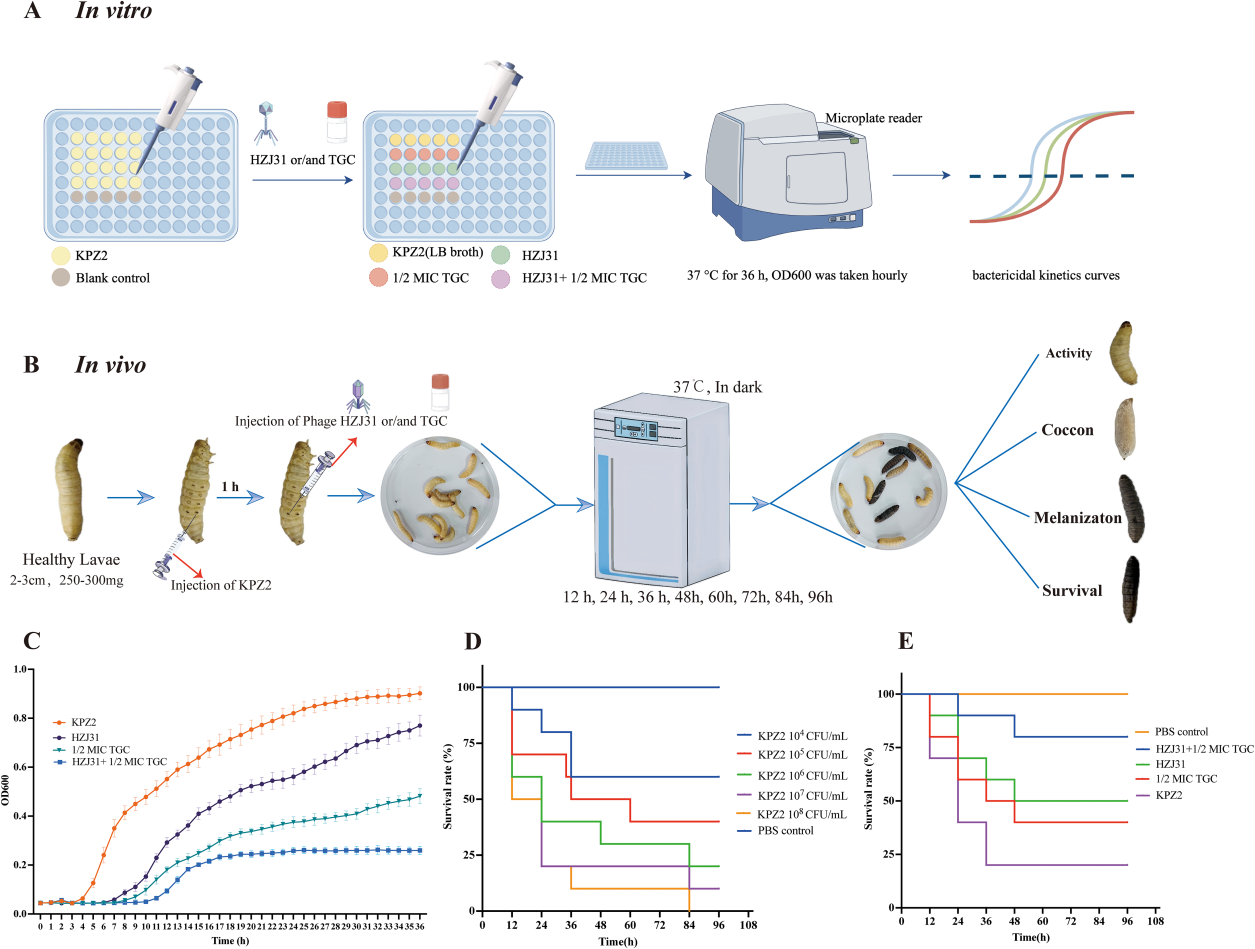
Antibacterial efficacy of phage HZJ31 and TGC against KPZ2 in Vivo and in Vitro. **(A)** Schematic of the *in vitro* bactericidal kinetics assay. KPZ2 was cultured in a 96-well plate and treated with phage HZJ31 (MOI = 0.001), 1/2 MIC TGC, or their combination. OD600 values were measured hourly over 36 hours at 37 °C using a microplate reader to generate bactericidal kinetics curves. **(B)** Schematic of the bactericidal efficacy in the *G.mellonella* infection model. Larvae were injected with KPZ2 (10 μL) into the last left proleg and incubated for 1 h, followed by injection of phage HZJ31, 1/2 MIC TGC, or their combination into the last right proleg. Larvae were monitored at 12-h intervals for survival over 96 hours. **(C)** *In vitro* bactericidal activity of phage HZJ31 and TGC against KPZ2 strain, mean ± SD. **(D)** Survival curves of *G.mellonella* larvae injected with different concentrations of KPZ2 to determine the optimal bacterial inoculum. An inoculum of 1×10^7^ CFU/mL resulted in approximately 80% mortality at 24 h, mean ± SD. **(E)** Therapeutic efficacy of HZJ31 and TGC in *G.mellonella* infection model, mean ± SD.

The schematic diagrams of the in vitro bactericidal kinetics assay and the G. mellonella infection model are shown in **Figures 3A** and **3B**, respectively. In this study, the combined antibacterial effect of phage HZJ31 and TGC was first evaluated *in vitro*. Prior to combination testing, the MIC of TGC against strain KPZ2 was determined to be 1.0 μg/mL. Then the bactericidal kinetics of phage HZJ31(MOI = 0.001), TGC (1/2 MIC, 0.5 μg/mL), and their combination against strain KPZ2 were evaluated over a 36-h period (**Figure 3C**). During the initial 6 h, the bacterial growth among all groups was generally consistent, with no significant differences in OD600. In the HZJ31 group, although the OD600 values gradually increased after 7 h, they remained significantly lower than those of the KPZ2 group throughout the entire experiment. In the 1/2 MIC TGC group, bacterial growth was also delayed, with OD600 increasing at a slower rate than in the HZJ31 group, reaching approximately 0.45 at 36 h. In the HZJ31 combined with the 1/2 MIC TGC group, no significant increase in OD600 was observed during the first 10 h, followed by a slight increase thereafter. However, the OD600 of the HZJ31 with 1/2 MIC TGC group remained at a low level (< 0.3) throughout the 36-h period, significantly lower than those in all other groups (*P* < 0.05), indicating a synergistic antibacterial effect of the combined treatment.

Given the significant similarities between the innate immune responses of *G.mellonella* larvae and vertebrates, the *G.mellonella* model is widely used as an alternative to evaluating bacterial virulence and *in vivo* antimicrobial efficacy. We then evaluated the bactericidal efficacy of phage HZJ31 and TGC in the *G.mellonella* infection model. In this study, we established an infection model by injecting *G.mellonella* larvae with varying concentrations of bacteria, determining that an optimal inoculum of 10^7^ CFU/mL for KPZ2 resulted in an 80% mortality rate at 24 h (**Figure 3D**). Significant differences were observed in the survival curves among the groups (log-rank test, *P* < 0.05). Compared to the KPZ2 group, the survival rates at 96 h were 50% and 60% for the phage HZJ31 and TGC treatments, respectively, while the survival rate for larvae treated with the combination of phage HZJ31 and TGC was 70% (**Figure 3E**). In summary, phage HZJ31 effectively controlled KPZ2 infection *in vivo.*

### 3.4 The antibiofilm activity of phage HZJ31

**Figure 4:**
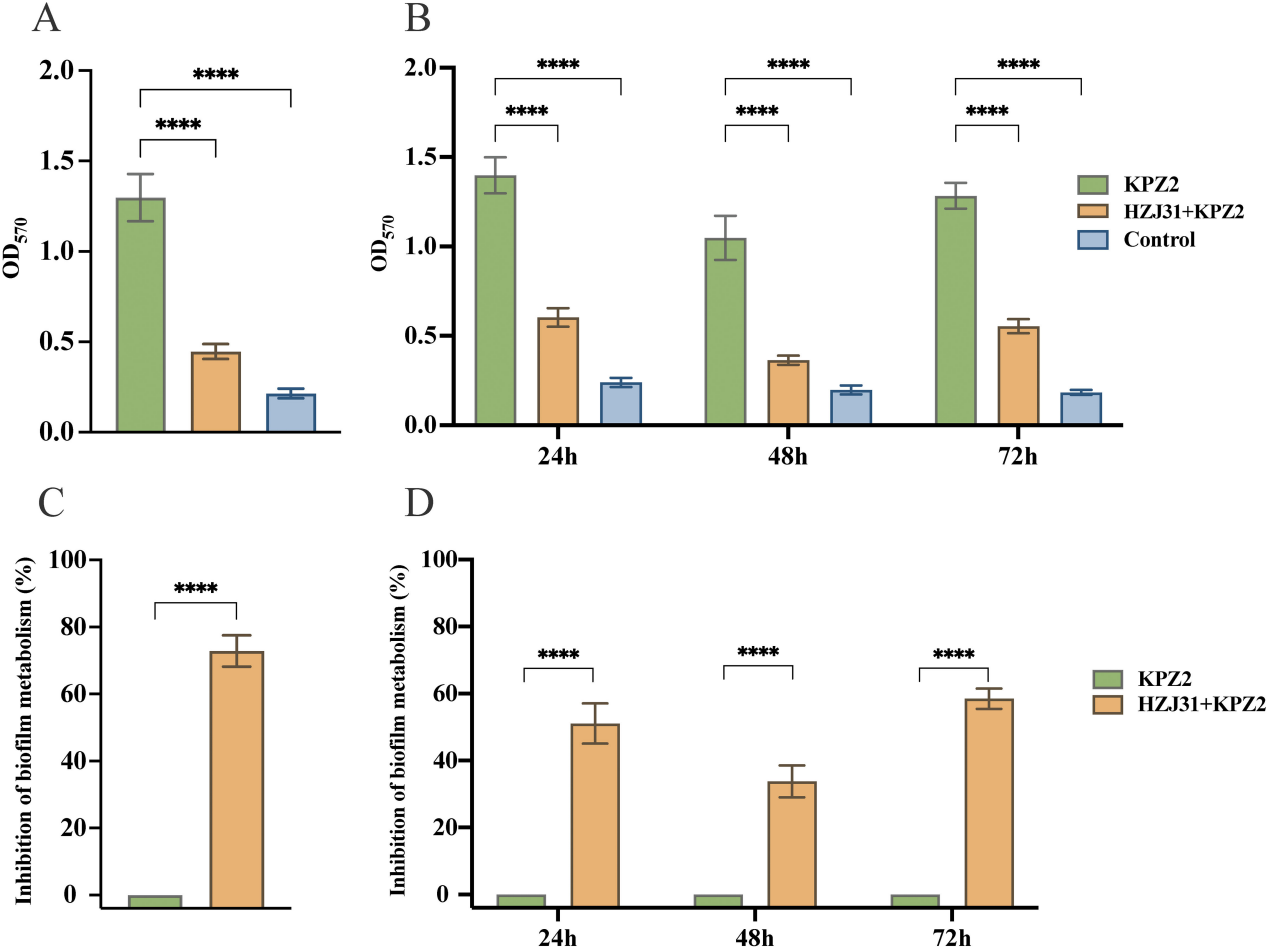
The antibiofilm activity of phage HZJ31.The crystal violet assay was used to quantify biofilm biomass and assess the disruptive effect of phage HZJ31 on pre-formed biofilms **(A)**and its inhibitory effect on biofilm formation **(B).** The XTT assay measured bacterial viability within biofilms to assess the disruptive **(C)** and inhibitory effects **(D)** of phage HZJ31. All data are shown as mean ± SD. ****P <0.0001.

The results showed that phage HZJ31 significantly inhibited KPZ2 biofilm formation at 24 h, 48 h, and 72 h (*P* < 0.05, **Figure 4A**). The inhibitory effect of phage HZJ31 on bacterial growth within the KPZ2 biofilm was assessed, showing a 59.45% inhibition rate at 72 h (*P* < 0.05) (**Figure 4C**). When phage HZJ31 was co-cultured with preformed KPZ2 biofilm for 6 h, significant disruption of the biofilm was observed (**Figure 4B**), with bacterial inhibition within the biofilm exceeding 70% (*P* < 0.05, **Figure 4D**). These findings demonstrate that phage HZJ31 effectively inhibits the formation of KPZ2 biofilms and disrupts preformed biofilms, exerting a substantial inhibitory effect on the bacteria within the biofilm.

### 3.5 Fitness cost of phage resistance in KPZ2-R: impaired capsule, growth, and virulence

**Figure 5:**
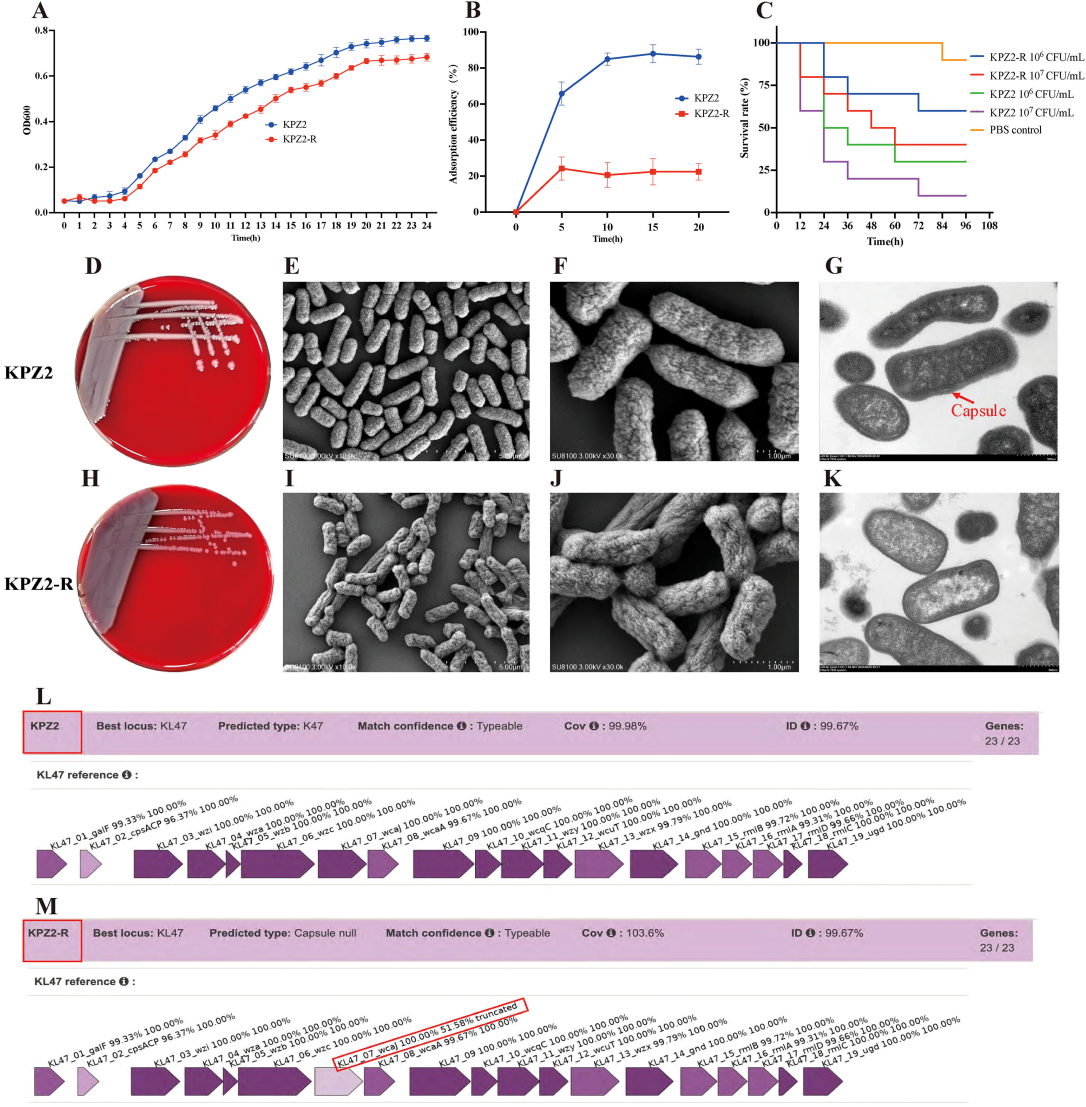
Characterization of phage-resistant strain KPZ2-R. **(A)** Growth curves of KPZ2 and KPZ2-R in LB broth measured by OD600, mean ± SD. **(B)** Comparison of adsorption efficiency between KPZ2 and KPZ2-R, mean ± SD. **(C)** Comparison of virulence between KPZ2 and KPZ2-R in the *G.mellonella i*nfection model. **(D, H)** Colony morphology of the strain KPZ2 and KPZ2-R on blood agar plates. **(E, F, I, J)** SEM images of KPZ2 **(E, F)** and KPZ2-R **(I , J)**. KPZ2 showed a smooth, dense, and uniform surface with a thick structure. In contrast, KPZ2-R had a rough, uneven surface with wrinkles or depressions and a thinner structure, along with evident capsule defects. **(G, K)** TEM images of KPZ2 **(G)** and KPZ2-R **(K)**. KPZ2 exhibited a well-defined, prominent capsule surrounding the cell, whereas KPZ2-R displayed a significantly reduced and poorly defined capsule. **(L, M)** Kaptive-based capsular locus analysis. Both strains matched the KL47 capsular type with 99.67% identity. KPZ2 showed complete locus coverage (99.98%) with intact capsule biosynthesis genes, while KPZ2-R was classified as “capsule null” due to a truncated wcaJ gene (51.58% identity).

After 24 h of co-culturing KPZ2 with phage HZJ31 (MOI = 0.001), the culture appeared visibly turbid, suggesting the emergence of phage-resistant mutants. Subsequent plating on LB agar and purification on blood agar yielded colonies that were no longer susceptible to phage HZJ31, as confirmed by both the double-layer agar method and spot assay, which showed no lysis zones. Mass spectrometry analysis identified all resistant colonies as *Klebsiella pneumoniae*, excluding the possibility of contamination. One phage-resistant isolate (designated KPZ2-R) was randomly selected from these colonies for further comparison with the wild-type strain. KPZ2 exhibited a slightly faster growth rate in LB broth than KPZ2-R, as measured by OD600 (**Figure 5A**). The phage adsorption efficiency of KPZ2 was 65.82% at 5 min, significantly higher than that of KPZ2-R (24.24%), and continued to increase over time, reaching 88.00% at 15 min. Conversely, the adsorption efficiency of KPZ2-R remained low throughout the incubation period (**Figure 5B**).

We also compared the virulence of KPZ2 and KPZ2-R using the *G.mellonella* infection model. At 10^6^ and 10^7^ CFU/mL of KPZ2, the survival rates of larvae at 48 h were 40% and 20%, respectively, whereas larvae injected with the same doses of KPZ2-R had survival rates of 70% and 50% (**Figure 5C**). Overall, KPZ2-R demonstrated lower virulence than KPZ2 *in vivo*. We further compared the antibiotic susceptibility of KPZ2 and KPZ2-R (shown in **Table 3**). Among all antibiotics tested, KPZ2 was only susceptible to TGC, polymyxin B, and ceftazidime/avibactam, while resistant to the others. In contrast, KPZ2-R showed increased susceptibility, shifting from resistance (R) to susceptibility (S) to piperacillin and gentamicin.

**Table 3:** The antibiotic susceptibility of KPZ2 and KPZ2-R.

Antibiotics	KPZ2		KPZ2-R	
	MIC	S/I/R	MIC	S/I/R
Tigecycline	≤1	S	≤1	S
Polymyxin B	≤0.05	S	≤0.05	S
Ceftazidime-avibactam	≤4	S	≤4	S
Piperacillin	≥128	R	≤32	S
Gentamycin	≥16	R	≤4	S
Phosphomycin	≥64	R	≥64	R
Ceftazidime	≥64	R	≥64	R
Ampicillin	≥32	R	≥32	R
Tobramycin	≥16	R	≥16	R
Imipenem	≥16	R	≥16	R
Ciprofloxacin	≥4	R	≥4	R
Cefuroxime sodium	≥64	R	≥64	R
Levofloxacin	≥8	R	≥8	R
Cefazolin	≥64	R	≥64	R
Cefoperazone/Sulbactam	≥64	R	≥64	R
Meropenem	≥16	R	≥16	R
Cotrimoxazole	≤20	S	≤20	S
Amikacin	≥64	R	≥64	R
Ceftriaxone	≥64	R	≥64	R
Cefotetan	≥64	R	≥64	R
Piperacillin/tazobactam	≥128	R	≥128	R
Ampicillin/sulbactam	≥32	R	≥32	R
Aztreonam	≥64	R	≥64	R
Cefuroxime	≥64	R	≥64	R

S, Sensitivity; I, Intermediary; R, Resistance.

On blood agar, KPZ2 formed large, smooth, plump, and moist colonies with a milky white appearance (**Figure 5D**), while the phage-resistant mutant KPZ2-R formed smaller, gray, semi-transparent colonies, but no obvious difference in diameter (**Figure 5H**). SEM revealed that the surface of KPZ2 appeared smooth, dense, and uniform, exhibiting a full and thick structure without visible indentations (**Figures 5E, F**). In contrast, KPZ2-R displayed a rough, uneven surface with wrinkles or depressions, a comparatively thinner structure, and showed evident structural defects in the capsule (**Figures 5I, J**). TEM further confirmed these differences, revealing a prominent, well-defined capsule surrounding KPZ2 (**Figure 5G**), while KPZ2-R exhibited a significantly reduced and poorly defined capsule with only a faint or nearly absent surrounding layer (**Figure 5K**), indicating impaired capsule formation in the resistant mutant.

Consistent with these observations, Kaptive-based analysis revealed that both KPZ2(GenBank: CP178862.1) and its phage-resistant mutant KPZ2-R (GenBank: CP192283.1) matched the KL47 capsular locus with 99.67% sequence identity (**Figures 5L, M**). KPZ2 exhibited complete locus coverage (99.98%) and intact capsule biosynthesis genes. However, KPZ2-R was classified as “capsule null” due to a truncated *wcaJ* gene (51.58% identity), suggesting the loss of capsule synthesis capability is associated with phage resistance.

## 4 Discussion

In recent years, CRKP has rapidly spread worldwide, resulting in high mortality rates and limited treatment options, and has become a serious public health concern (Karampatakis et al., 2023). Therefore, there is an urgent need to explore alternative therapeutic strategies (Chang et al., 2022). Amid the growing challenge of antimicrobial resistance, phage therapy has emerged as a promising antibacterial approach (Hatfull et al., 2022; Kou et al., 2024). Numerous preclinical studies and clinical case reports have demonstrated the therapeutic potential of phages in treating KP infections (Kou et al., 2024; Xing et al., 2025).

Phage HZJ31 demonstrated stability across pH values ranging from 5 to 9 and temperatures from 4°C to 50°C. This robust stability profile facilitates its activity under diverse environmental conditions, simplifying storage and transportation logistics. Following a 48 co-culture of phage HZJ31 with THP-1 cells, the CCK-8 assay yielded no detectable cytotoxicity, indicating biocompatibility. Genomic sequencing further disclosed an absence of genes conferring toxicity or antibiotic resistance, offering initial assurance of its safety for therapeutic applications. These findings underpin the promising clinical potential of phage HZJ31.

Biofilms significantly contribute to recurrent medical device-associated chronic infections and play a pivotal role in bacterial adaptive resistance and evasion of host immune surveillance (Chadha et al., 2022). These complicated microbial communities are composed of bacteria that are embedded within a self-produced matrix of extracellular polymers (EPS) consisting of extracellular polysaccharides, proteins, and extracellular DNA (eDNA) (Flemming et al., 2016). The biofilm-forming ability of KP is closely associated with the expression of virulence factors such as capsular polysaccharides and outer membrane proteins (Sauer et al., 2022) (Li et al., 2024). KP strains with robust biofilm-forming capabilities exhibit heightened virulence, suggesting more severe infections and adverse outcomes (Zheng et al., 2018; Tang et al., 2024). Phage therapy has emerged as a promising strategy to combat biofilm-associated infections. In this study, phage HZJ31 effectively inhibited KPZ2 biofilm formation at 24 h, 48 h, and 72 h and compromised the structural integrity of preformed biofilms, resulting in efficient bacterial lysis within the biofilms.

Phages can encode enzymes, such as depolymerase, endolysin and holin, that degrade biofilm matrices, including polymers and capsular polysaccharides, thus facilitating the disruption of bacterial biofilm integrity, which is a key mechanism of phage action against bacterial biofilms (Lin et al., 2014; Topka-Bielecka et al., 2021; Lendel et al., 2024; Zhao et al., 2024a). The effectiveness of HZJ31 in overcoming bacterial biofilm defenses presents a new avenue for the treatment of refractory infections. A total of 48 ORFs were identified in the genome of phage HZJ31 through Prokka annotation, of which 33 were functionally characterized. Notably, ORF 20 and ORF 39 were predicted to encode a lysin and a holin protein, respectively. In addition, 15 ORFs were annotated as hypothetical proteins, suggesting that some of them may encode previously uncharacterized enzymes involved in biofilm matrix degradation. Further structural and functional analyses are required to elucidate their potential roles.

Phage-antibiotic combination therapy emerges as a promising strategy for combating antibiotic-resistant infections. This therapeutic approach harnesses the distinct antimicrobial mechanisms of phages and antibiotics to expand the host range, augment antibacterial potency, and retard the development of phage resistance. Furthermore, it has the potential to diminish the necessary antibiotic dosages, alleviate associated side effects, and optimize treatment efficacy in complex infections (Van Nieuwenhuyse et al., 2021; Ashworth, 2024). In our study, tigecycline was selected for combination therapy based on susceptibility testing, which showed that KPZ2 was resistant to most antibiotics but remained sensitive to TGC, polymyxin B, and ceftazidime-avibactam (**Table 3**). TGC, a tetracycline derivative, manifests broad-spectrum antimicrobial properties and an extended half-life by impeding protein synthesis in a spectrum of pathogens, including antibiotic-resistant bacteria and biofilms (Giamarellou and Poulakou, 2011). Both monotherapy of TGC and its synergistic combinations with other antibiotics have shown promising treatment efficacies against CRKP infections (Park et al., 2024; Xie et al., 2024). In this study, the bactericidal kinetics data revealed that phage HZJ31 and TGC exhibited distinct antimicrobial effects when applied individually or in combination against KPZ2. The phage HZJ31 group demonstrated a moderate inhibitory effect, with OD600 values rising after 7 h but remaining significantly lower than those of the untreated control throughout the 36-h observation period. This indicates that phage HZJ31 can delay bacterial proliferation and partially suppress growth, though phage resistance may emerge over time. Notably, the combination of phage HZJ31 with 1/2 MIC TGC achieved superior bacterial suppression, suggesting a synergistic interaction between phage HZJ31 and sub-inhibitory concentrations of TGC in both augmenting bactericidal efficacy and delaying the emergence of resistant strains. In addition to TGC, other antibiotics such as polymyxin B, colistin, and ceftazidime-avibactam may also be considered for phage-antibiotic combination strategies (Wang et al., 2021; Han et al., 2022; Cristinziano et al., 2024), depending on the antibiotic susceptibility profile and infection context (Diallo and Dublanchet, 2022) (Morrisette et al., 2020; Zhao et al., 2024b).

In the *G.mellonella* infection model, larvae treated with phage HZJ31 or TGC alone had survival rates of 50% and 60%, respectively, after 96 h, while the HZJ31-TGC combination exhibited a significantly improved survival rate (70%). These results demonstrate that the phage HZJ31-TGC combination enhances antibacterial activity both *in vitro and in vivo*. Furthermore, the phage-antibiotic combination exhibited synergistic effects in biofilm degradation. The therapeutic efficacy of phage-antibiotic combination therapy is contingent upon a multitude of factors, such as the specific types of phages and antibiotics employed, the order of administration, dosages, and the duration of treatment. It is noteworthy that certain phage-antibiotic combinations may exhibit antagonistic interactions. In light of the absence of standardized protocols for phage-antibiotic therapy, there is an imperative need for systematic experimentation to ascertain the most efficacious combinations. These experiments should also carefully monitor the emergence of resistant strains during treatment. Highly personalized treatment regimens should be designed to maximize therapeutic efficacy while minimizing adverse reactions (Hong et al., 2024) (Fungo et al., 2023).

In monophage therapy, bacteria rapidly develop resistance to phages under selective pressure, which limits the application of phage therapy (Strathdee et al., 2023; Laanto, 2024; Pal et al., 2024). The mechanisms of phage resistance are complex and diverse, including preventing phage adsorption, blocking phage DNA injection, cleaving the phage genome, abortive infection, and quorum sensing, among others (Laanto et al., 2020). Adsorption is the initial step of phage infection of bacteria and a crucial factor determining the success of phage therapy. Bacteria can block phage adsorption through several mechanisms: mutation or modification of the phage receptor, extracellular matrix interference with the receptor, and competitive inhibition by receptor analogs (Laanto et al., 2020). Capsular polysaccharides, outer membrane proteins, and lipopolysaccharides serve as the adsorption receptors for KP phages, and their loss or mutation can lead to phage resistance (Camprubí, 1991; Henrici De Angelis et al., 2021; Cai et al., 2022; Fang and Zong, 2022; Geng et al., 2023). The capsule often masks the bacterial receptors, preventing direct binding between the phage and the receptor (Samson et al., 2013). In this study, we observed that KPZ2 developed phage resistance under selective pressure from phage HZJ31 by losing its capsule, resulting in a significant decrease in the phage’s adsorption efficiency to the resistant mutant KPZ2-R. Moreover, we observed a significant trade-off phenomenon — KPZ2-R exhibited altered colony morphology, slower growth rate, and reduced virulence, which could increase its susceptibility to immune clearance during actual infections. Interestingly, KPZ2-R regained sensitivity to piperacillin and gentamicin, a phenomenon not uncommon in bacterial resistance mechanisms (Hu et al., 2024b). Although bacteria may escape phage infection by altering or shedding their capsule and outer membrane proteins, this adaptation can inadvertently compromise their defense against antibiotics, consequently rendering them susceptible once again (Gordillo Altamirano et al., 2021; Nang et al., 2024). The employment of a phage cocktail comprising both capsule-dependent and -independent phages, or a strategic sequence of phage and antibiotic administration, may yield superior therapeutic outcomes, offering innovative strategies and perspectives for phage therapy.

This study has several limitations that should be acknowledged. First, the host range of phage HZJ31 was assessed using 30 clinical CRKP isolates through spot assays. The limited number of isolates may not fully represent the genetic and phenotypic diversity of CRKP in clinical settings. Moreover, most strains were collected from a single hospital, raising the possibility of strain clustering and potential bias toward nosocomial infection types. Future studies will include a broader and more diverse set of CRKP isolates from multiple geographic and clinical sources to enhance the accuracy and generalizability of the host range evaluation. Second, the antibacterial and antibiofilm effects of phage HZJ31 were evaluated only against a single CRKP strain, without investigating potential cross-species or cross-strain activity. Additionally, the synergistic antibacterial activity was tested only in combination with TGC, to which the tested strain was already susceptible. Expanding the evaluation to include multiple antibiotics and additional strains will provide a more comprehensive understanding of its synergistic potential. The *in vivo* antibacterial efficacy was limited to the *G.mellonella* model and has not yet been validated in advanced mammalian models, which restricts the generalizability of the study. Lastly, the biosafety of phage HZJ31 was only preliminarily assessed at the genetic and cellular levels. Further evaluation in mammalian models is necessary to comprehensively assess its safety and therapeutic efficacy.

In conclusion, we successfully isolated a novel lytic phage, HZJ31, which has demonstrated high environmental stability and biocompatibility. Furthermore, the synergistic application of HZJ31 with TGC showed a markedly enhanced antibacterial activity in both *in vitro* and the *G.mellonella* infection model. These findings provide theoretical support for the potential clinical application of phage HZJ31.

## Data Availability

The datasets presented in this study can be found in online repositories. The names of the repository/repositories and accession number(s) can be found in the article/supplementary material.
